# Draft genome of six Cuban *Anolis* lizards and insights into genetic changes during their diversification

**DOI:** 10.1186/s12862-022-02086-7

**Published:** 2022-11-04

**Authors:** Shunsuke Kanamori, Luis M. Díaz, Antonio Cádiz, Katsushi Yamaguchi, Shuji Shigenobu, Masakado Kawata

**Affiliations:** 1 Graduate School of Life Sciences, Tohoku University, Sendai, Japan; 2 National Museum of Natural History of Cuba, Havana, Cuba; 3 Faculty of Biology, University of Havana, Havana, Cuba; 4 Trans-Omics Facility, National Institute for Basic Biology, Okazaki, Japan; 5 Department of Basic Biology, School of Life Science, The Graduate University for Advanced Studies, SOKENDAI, Okazaki, Japan; 6 Department of Biology, University of Miami, Coral Gables, USA

**Keywords:** Anole, Comparative genomics, Repeat elements, Gene duplication, Effective population size

## Abstract

**Background:**

Detecting genomic variants and their accumulation processes during species diversification and adaptive radiation is important for understanding the molecular and genetic basis of evolution. *Anolis* lizards in the West Indies are good models for studying evolutionary mechanisms because of the repeated evolution of their morphology and the ecology. We performed de novo genome assembly of six Cuban *Anolis* lizards with different ecomorphs and thermal habitats (*Anolis isolepis*, *Anolis allisoni*, *Anolis porcatus*, *Anolis allogus*, *Anolis homolechis*, and *Anolis sagrei*). We carried out a comparative analysis of these genome assemblies to investigate the genetic changes that occurred during their diversification.

**Results:**

We reconstructed novel draft genomes with relatively long scaffolds and high gene completeness, with the scaffold N50 ranging from 5.56 to 39.79 Mb and vertebrate Benchmarking Universal Single-Copy Orthologs completeness ranging from 77.5% to 86.9%. Comparing the repeat element compositions and landscapes revealed differences in the accumulation process between Cuban trunk-crown and trunk-ground species and separate expansions of several families of LINE in each Cuban trunk-ground species. Duplicated gene analysis suggested that the proportional differences in duplicated gene numbers among Cuban *Anolis* lizards may be associated with differences in their habitat ranges. Additionally, Pairwise Sequentially Markovian Coalescent analysis suggested that the effective population sizes of each species may have been affected by Cuba’s geohistory.

**Conclusions:**

We provide draft genomes of six Cuban *Anolis* lizards and detected species and lineage-specific transposon accumulation and gene copy number changes that may be involved in adaptive evolution. The change processes in the past effective population size was also estimated, and the factors involved were inferred. These results provide new insights into the genetic basis of *Anolis* lizard diversification and are expected to serve as a stepping stone for the further elucidation of their diversification mechanisms.

**Supplementary Information:**

The online version contains supplementary material available at 10.1186/s12862-022-02086-7.

## Background

Elucidating how genetic variation emerges and accumulates during species diversification is important for understanding the mechanisms that create species diversity and adaptive evolution. With the recent development of sequencing technologies, the genomes of taxonomic groups other than model organisms like humans and mice have been sequenced. In some lineages, the genomes of many species have been sequenced and assembled, and progress has been made in elucidating the emergence and accumulation processes of mutations, leading to a better understanding of adaptive evolution and diversity generation within lineages [1–4]. Parallel evolution and adaptive radiation are important for investigating the mechanisms of species diversification and adaptive evolution. In particular, in Galapagos finches and African cichlids, good models of species diversification due to their parallel evolution and adaptive radiation, genomic analyses for many species have facilitated elucidation of the genetic mechanisms that enabled diversification and repeated adaptive evolution [1, 2, 4].

*Anolis* lizards of the West Indies are model organisms that underwent parallel evolution and adaptive radiation. Phylogenetically distant species with similar structural habitats often have similar morphology and behavior [5], and adaptive evolution of morphology and behavior to these structural habitats occurred multiple times independently [6, 7]. The common physical structures, morphology, and behavior are called “ecomorphs.” Ecomorphs of *Anolis* lizards in the West Indies include crown-giant, trunk-crown, twig, trunk, trunk-ground, and grass-bush. Crown-giant species are large, inhabit the canopy and branches high up in trees, and have a low movement rate; trunk-crown species have numerous toepad lamellae that aid climbing, live on tree trunks and branches, and have a high movement rate; twig species have a short tail and limbs, use thin branches, and have a low movement rate; trunk species are found on tree trunks and have a short tail and a high movement rate; trunk-ground species inhabit tree trunks and the ground, have a long tail and hind limbs, jump, and have a low movement rate; grass-bush species inhabits low, thin structures such as bushes, have a very long tail and a slender body, jump, and have a low movement rate [5]. Moreover, their thermal habitats are diverse, and there is a high diversity in the body temperature among these species [5, 8, 9]. Differences in thermal habitats often occur between closely related species [9–13], suggesting that adaptive evolution to various thermal habitats has also happened repeatedly. Therefore, elucidating the creation and accumulation processes of genetic mutations among *Anolis* lizards in the West Indies is expected to yield insights into the genetic or molecular basis of their morphological features, physiology, and ecology, the mechanisms and repeatability of evolution, and the relationship between genetic mutation, evolvability, and evolutionary constraints.

The genome assembly of *Anolis carolinensis*, the only species of *Anolis* lizard native to the United States, has already been reconstructed [14]. The *A. carolinensis* genome and its annotation information have been used to elucidate the genetic mechanisms of environmental adaptation and ecomorph evolution in *Anolis* lizards [15–20]. Furthermore, the genome assemblies of three Central American *Anolis* lizards (*Anolis frenatus*, *Anolis auratus*, and *Anolis apletophallus*) have also been reconstructed [21]. Comparative analyses of these genomes revealed the positive selection of many genes with functions involved in adaptively evolved traits [21]. Moreover, a highly complete chromosome-scale genome assembly of *Anolis sagrei ordinatus*, a subspecies of *A. sagrei* collected in the Bahamas, has recently been reported [22]. Like *A. carolinensis*, *A. sagrei* is an excellent model among *Anolis* lizards for evolutionary and ecological studies, and further advances in the genetic basis of adaptive evolution, plasticity, and species invasiveness through comparative analyses among populations are expected. Moreover, the *hox* gene cluster sequence and expression have been analyzed for many *Anolis* lizards [23, 24]. However, the genome assembly of most *Anolis* lizards of the West Indies, a good model for studying convergent evolution and adaptive radiation, has not been reconstructed, and little is known about the emergence and accumulation processes of genetic variation other than point mutations and genetic variations in non-coding regions, during adaptive evolution.

Cuba hosts 65 species of *Anolis* lizards, the highest number in the West Indies islands. Their ecomorphs and thermal habitats are diverse, with species belonging to all six ecomorphs and unique ecomorphs [5, 25]. Species belonging to the same ecomorph construct monophyletic lineages [25]. Closely related species within monophyletic lineages have different thermal habitats with varying tree cover, air temperatures, and degrees of exposure to sunlight [10–13, 25]. For example, three trunk-ground ecomorph species are closely related: *Anolis allogus*, *A. sagrei*, and *Anolis homolechis*. *Anolis allogus* inhabits relatively cool forests and does not bask; *A. sagrei* inhabits open areas outside forests, where there is more sun, and frequently basks under direct sunlight; and *A. homolechis* inhabits forest margins, where temperatures are intermediate, and basks under filtered sun [10–13, 25]. Furthermore, the degree to which these lizards thrive in urban areas varies by species. Some species are rarely found in urban areas, while *Anolis porcatus*, *Anolis allisoni*, and *A. sagrei*, which naturally inhabit hot and open areas, thrive in urban regions [11, 12, 26]. Several species of *Anolis* lizard have been introduced to and colonized regions other than their native areas. For instance, *A. sagrei*, native to Cuba, has invaded Florida and Taiwan [27], and *A. carolinensis*, a close relative of *A. porcatus* that is native to Cuba, has invaded Okinawa, the Ogasawara Islands, and the Hawaii Islands from its native habitat on mainland USA [28, 29]. In *Anolis* lizards of the West Indies, species that experience hot, dry conditions in their natural habitat may be more tolerant of urban environments [26]. Therefore, elucidating the genetic variation among Cuban *Anolis* lizards will help reveal the adaptive evolutionary mechanisms of ecomorphs, thermal habitats, urban tolerance, and invasiveness. In Cuban *Anolis* lizards, the evolutionary mechanisms of adaptation to thermal habitats have been studied at the genetic level by comparing three trunk-ground species, *A. allogus*, *A. homolechis*, and *A. sagrei*, that inhabit different thermal habitats. Akashi et al. (2016) [30] analyzed the transcriptomic expression variation with differences in rearing temperature. Akashi et al. (2018) [31] also investigated the temperature-triggered escape behavior from heat and the activation of a molecular heat sensor. Moreover, a study have carried out a phylogenetic analysis of the coding sequences of several Cuban *Anolis* lizards and identified genes that underwent positive selection in *A. allisoni*, *A. porcatus*, and *A. sagrei*, which are adapted to open areas and thrive in urban areas [32]. Recently, genome editing technology has been established for *A. sagrei* [33], accelerating our understanding of the genetic basis of adaptive evolution in *Anolis* lizards. However, the genome assembly of the Cuban *Anolis* lizards has not been reconstructed, and most genetic variations involved in adaptive evolution remain undetected.

In this study, we reconstructed novel draft genome assemblies of six species of Cuban *Anolis* lizards: three closely related trunk-crown species (the forest-dwelling *Anolis isolepis* and the hot, open area-dwelling *A. allisoni* and *A. porcatus*) and three closely related trunk-ground species (the forest-dwelling *A. allogus*, the hot, open area-dwelling *A. sagrei*, and the forest margin-dwelling *A. homolechis*) and investigated their differences in genomic characteristics, such as the gene and transposable element (TE) composition, between these species. Although the genome assembly of *A. sagrei ordinatus* from the Bahamas, a subspecies of *A. sagrei*, has been reported [22], *A. sagrei* originated in Cuba [34]. Hence, we analyzed the genome assembly of the Cuban *A. sagrei* (*A. sagrei sagrei*) to unify the reconstruction and annotation methods of these genome assemblies and detect genetic variations during the formation of common ecological *A. sagrei* features acquired before their introduction to other regions. Additionally, the change processes of the past effective population sizes of each species were estimated. Results from analyses of these novel draft genome assemblies will provide essential information to further elucidate the diversification and adaptive evolution mechanisms of *Anolis* lizards.

## Results

### de novo genome assembly and gene annotation

**Fig. 1:**
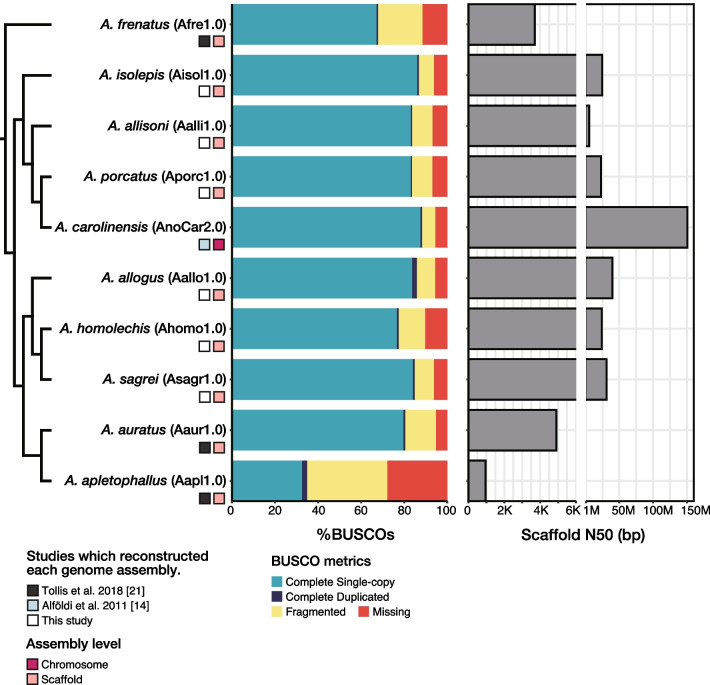
Phylogenetic relationships between the *Anolis* lizards included in this study and descriptive statistics of their genome assemblies

The genomes of *A. isolepis*, *A. allisoni*, *A. porcatus*, *A. allogus*, *A. homolechis*, and *A. sagrei* were sequenced at 47.26–63.30 × coverage (Table 1). The genome sizes estimated using Supernova v.2.1.1 [35] were 2.05–2.66 Gb (Table 1). de novo genome assembly, removing haplotigs and overlaps, resulted in a scaffold N50 and a total length of 5.56–39.79 Mb and 1.53–1.88 Gb, respectively. The coverage of complete vertebrate Benchmarking Universal Single-copy Orthologs (BUSCOs) and duplicated vertebrate BUSCOs were 77.5–86.9% and 0.6–2.2%, respectively (Table 2). Haplotig and overlap removal resulted in a slight reduction in the scaffold N50, complete vertebrate BUSCO coverage (Table 2; Additional file 1: Table S1), and approximately half of the duplicated vertebrate BUSCO coverage (Additional file 1: Table S2). The scaffold N50 of these genome assemblies were much shorter than the chromosome-level genome assembly of *A. carolinensis* (AnoCar2.0) but considerably longer than the draft genome assemblies of *A. frenatus* (Afren1.0), *A. auratus* (Aaur1.0), and *A. apletophallus* (Aapl1.0) (Fig. 1). Furthermore, the BUSCO coverage was comparable to that of the *A. carolinensis* genome (Anocar2.0) (Fig. 1).

**Table 1:** Genome sequencing results

Species	Total read data (Gb)	Coverage	Coverage after adjusting the number of reads	Estimated genome size (Gb)
*A. isolepis*	128	61.70 ×	56.64 ×	2.08
*A. allisoni*	129	57.34 ×	56.27 ×	2.26
*A. porcatus*	134	63.30 ×	56.80 ×	2.11
*A. allogus*	125	54.16 ×		2.33
*A. homolechis*	125	47.26 ×		2.66
*A. sagrei*	123	60.18 ×	56.48 ×	2.05

**Table 2:** Descriptive statistics of the reconstructed genome assemblies

Species	Scaffold N50 (Mb)	Total length (Gb)	Number ≧ 10 Kb	BUSCO metrics				GC content (%)
				Complete	(Single	Duplicated)	Fragmented	
*A. isolepis* (Aisol1.0)	24.70	1.67	2.24 K	86.9%	86.1%	0.8%	7.0%	40.14
*A. allisoni* (Aalli1.0)	5.56	1.75	5.28 K	83.8%	83.1%	0.7%	9.4%	40.11
*A. porcatus* (Aporc1.0)	23.09	1.74	5.80 K	83.7%	83.1%	0.6%	9.4%	40.0
*A. allogus* (Aallo1.0)	39.79	2.09	4.68 K	86.0%	83.8%	2.2%	8.4%	41.05
*A. homolechis* (Ahomo1.0)	24.32	1.97	8.60 K	77.5%	76.6%	0.9%	12.3%	41.02
*A. sagrei* (Asagr1.0)	31.30	1.90	7.39 K	85.0%	84.0%	1.0%	8.9%	40.96

The vertebrate BUSCO database (odb9) was used for the assessment of gene completeness

**Fig. 2:**
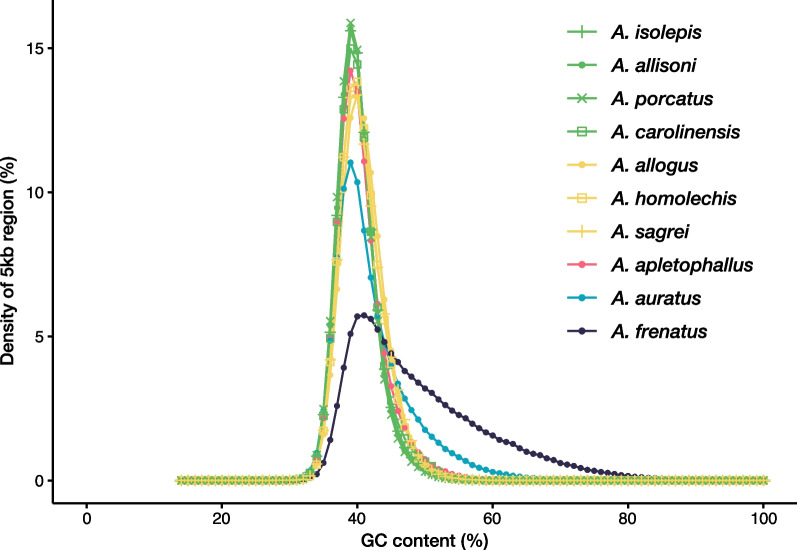
The distribution of GC content in 5-kb windows of genome assemblies for *Anolis* lizards. The green lines and points indicate the distributions of *A. isolepis*, *A. allisoni*, and *A. porcatus* from the Cuban trunk-crown lineage, and their relative, *A. carolinensis*; yellow lines and points indicate the distribution for *A. allogus*, *A. homolechis*, and *A. sagrei* from the Cuban trunk-ground lineage; navy blue, light blue, and gray lines and points indicate the distributions of *A. frenatus*, *A. auratus*, and *A. apletophallus,* respectively

The GC content in the genome assemblies of *A. isolepis*, *A. allisoni*, *A. porcatus*, *A. allogus*, *A. homolechis*, and *A. sagrei* after removing haplotigs and overlaps were 40.0–41.05% (Table 2). Those in the genome assemblies of mainland *Anolis* lizards are 40.32% for *A. carolinensis*, 49.44% for *A. frenatus*, 42.81% for *A. auratus*, and 40.92% for *A. apletophallus*. Although they were roughly equal, the GC content of Cuban trunk-ground lineage species was slightly higher than that of Cuban trunk-crown lineage species and their relative, *A. carolinensis*. Differences between these lineages were also observed in the frequency distribution of the GC content percentage within 5 kb windows with a gap of less than 50% (the GC content distribution) (Fig. 2). Although Tollis et al. (2018) [21] observed a higher GC bias in *A. frenatus* compared to the other three mainland species, comparison with six Cuban species verified that it had the highest GC content. Additionally, the GC content distribution was higher than that in the other species. For the other species, GC content distributions were created for each genome assembly after repeat masking. The GC content distributions after repeat masking differed less among the species or lineages than those before repeat masking (Additional file 1: Fig. S1).

Gene models were constructed on the repeat-masked genome assemblies after removing haplotigs and overlaps. Then, 21,688–25,839 protein-coding genes were predicted for six Cuban *Anolis* lizards (Table 3). The gene distributions are provided in Additional file 1: Fig. S2. The complete vertebrate BUSCO coverage for the predicted transcript sequences was 78.9–86.5% (Table 3).

**Table 3:** The results of gene prediction

Species	Predicted gene number	BUSCO metrics for transcript sequences of predicted genes			
		Complete	(Single	Duplicated)	Fragmented
*A. isolepis* (Aisol1.0)	21,688	84.8%	76.7%	8.1%	9.7%
*A. allisoni* (Aalli1.0)	23,503	83.0%	74.1%	8.9%	11.7%
*A. porcatus* (Aporc1.0)	24,100	81.8%	74.4%	7.4%	12.3%
*A. allogus* (Aallo1.0)	23,232	86.5%	77.5%	9.0%	9.2%
*A. homolechis* (Ahomo1.0)	25,839	78.9%	72.1%	6.8%	14.6%
*A. sagrei* (Asagr1.0)	23,819	84.2%	78.0%	6.2%	10.7%

Predicted gene numbers and BUSCO metrics for transcript sequences of predicted genes. The vertebrate BUSCO database (odb9) was used to assess gene completeness

### The composition of repeat elements and the repeat landscape

**Fig. 3:**
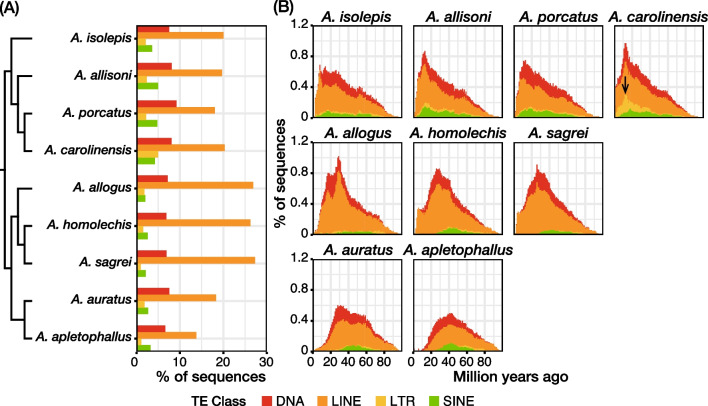
Repeat content and landscape of *Anolis* genomes. **A** Phylogenetic relationships between *Anolis* lizards included in the repeat element analysis and the sequence percentages of each transposable element (TE) class. **B** Repeat landscape for each TE class of *Anolis* lizards included in the repeat element analysis. The arrow points to a steep peak for LTR observed only in *A. carolinensis*

The percentage of repeat element lengths was similar for the six Cuban *Anolis* lizards at 36–41% (Additional file 1: Tables S3–S8). The repeat elements were reannotated by the same method for the genome assemblies of three mainland *Anolis* lizards (*A. carolinensis*, *A. auratus*, and *A. apletophallus*). The percentage of repeat element lengths in the *A. carolinensis*, *A. auratus*, and *A. apletophallus* genomes was 40%, 37%, and 27%, respectively (Additional file 1: Tables S9–S11). Then, we compared the composition and landscape of classes and families of the repeat elements among these nine species. The results indicated that the genomes of the three Cuban trunk-ground species (*A. allogus*, *A. homolechis*, and *A. sagrei*) had more LINEs than those of other species (Fig. 3; Additional file 1: Tables S3–S11). Additionally, the genome of *A. carolinensis* contained the most LTRs among the nine species (Fig. 3; Additional file 1: Tables S3–S11). Steep LTR waves were also observed only in the repeat landscape for *A. carolinensis* among these nine species. Among the three Cuban trunk-ground species, which had more LINEs, different accumulation waves were observed in the repeat landscapes of some LINE families (Additional file 1: Fig. S3). For example, the *A. homolechis* and *A. sagrei* genomes have more LINE-RTE-BovB than the *A. allogus* genome, and the two waves of its accumulation in *A. homolechis* and *A. sagrei* genomes were insignificant for *A. allogus* (Additional file 1: Fig. S3). Alternatively, the *A. apletophallus* genome, which is closely related to these three Cuban trunk-ground species, had considerably fewer LINEs among the nine species, which appears to be the primary reason for the remarkably low ratio of repeat element length to genome size (Fig. 3; Additional file 1: Tables S3–S11). *A. auratus* is closely related to the three Cuban trunk-ground species, but its genome has slightly fewer LINEs (Fig. 3; Additional file 1: Tables S3–S11). However, among the nine genomes, the percentage of simple repeats was very high (Additional file 1: Tables S3–S11).

### Analysis of gene number evolution

**Fig. 4:**
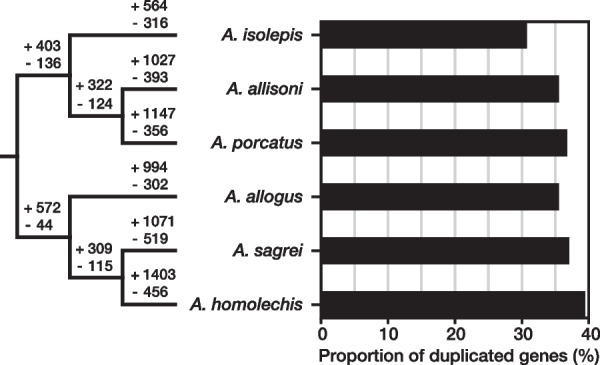
Phylogenetic relationships, the expansion or contraction in the number of genes in each branch, and the proportion of duplicated genes for the six Cuban *Anolis* lizards. The numbers above each branch of the phylogenetic tree represent the increase or decrease in the number of genes estimated in orthogroups, which includes genes from all species used in the analysis

The proportion of duplicated genes (*P*_D_: the number of duplicated genes/total number of genes) for *A. isolepis*, *A. allisoni*, *A. porcatus*, *A. allogus*, *A. homolechis*, and *A. sagrei* was 30.8–39.4% (Table 4, Fig. 4). The G-test of the independence of species and the number of duplicated and single genes with simultaneous test procedure (STP) detected associations between species and the number of duplicated and single genes (*P*-value = 8.2 × 10^−45^ for Cuban trunk-crown species and 2.1 × 10^−18^ for Cuban trunk-ground species) (Table 4). Furthermore, the test detected significant bounds on the ratio of duplicated gene numbers to single gene numbers between *A. isolepis* and *A. allisoni*, between *A. allisoni* and *A. porcatus* within the Cuban trunk-crown species (*P*-value = 0.014 and 1.2 × 10^−25^, respectively), between *A. allogus* and *A. sagrei*, and between *A. sagrei* and *A. homolechis* within the Cuban trunk-ground species (*P*-value = 5.2 × 10^˗7^ and 0.0021, respectively) (Table 4). Additionally, the analysis indicated that many gene number expansions and contractions occurred in the terminal branches of each species (Fig. 4).

**Table 4:**
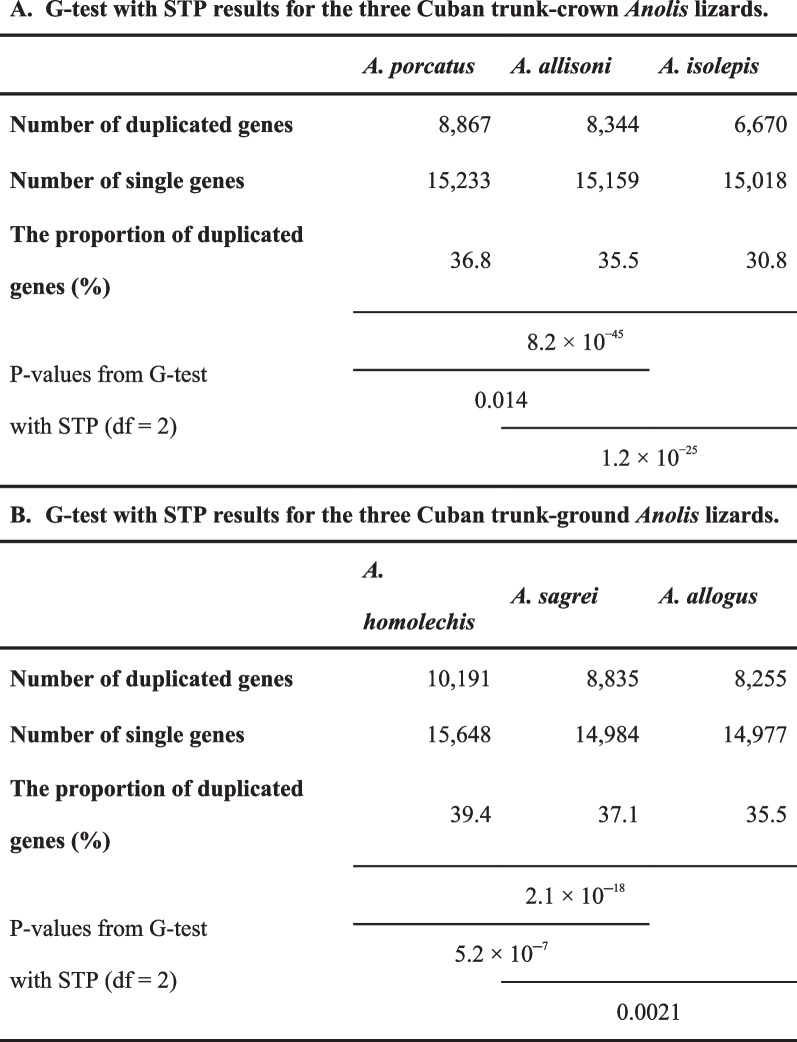
G-test with STP results for the homogeneity of the duplicated to single gene number ratio

G-test with STP results for the homogeneity of duplicated to single gene number ratio among (A) three Cuban *Anolis* lizards of the trunk-crown lineage and (B) three Cuban *Anolis* lizards of the trunk-ground lineage. P-values are from the results of the G-test with the degree of freedom (df) adjusted to 2 according to STP [6] performed among species, which are indicated by the black line

Next, we analyzed the enrichment of Gene Ontology (GO) terms of biological processes in ortholog groups (orthogroups) where gene number expansion or contraction occurred. Orthogroups that experienced gene number expansion in the *A. homolechis* terminal branch included significantly more orthogroups with the GO term “translation” (*P*-value adjusted with the Benjamini–Hochberg method = 0.0021). Most orthogroups with the GO term “translation” comprised genes encoding ribosomal proteins (Table 5).

**Table 5:** Orthogroups with the GO term “translation” and gene number expansion in *A. homolechis*

Orthogroup ID	The gene number of each species						Gene name of *A. homolechis*
	*A. isolepis*	*A. allisoni*	*A. porcatus*	*A. allogus*	*A. homolechis*	*A. sagrei*	
OG0000089	2	2	2	9	8	7	RPS2
OG0000513	2	2	2	3	4	3	RPL3, RPL3L
OG0000529	3	4	3	2	3	1	RPL6
OG0000580	2	2	3	3	4	2	RPS18
OG0000816	2	2	2	2	3	2	PHKA1, PHKA2
OG0000841	2	2	2	2	3	2	RPL27A
OG0000988	2	2	2	2	3	1	RPS6
OG0001300	2	2	2	1	3	2	RPL8
OG0001580	1	2	1	2	3	2	RPL9
OG0001594	1	2	1	2	3	2	RPL17
OG0001616	2	1	2	1	3	2	RPS5
OG0001671	1	1	1	1	5	2	RPL13A
OG0001716	1	3	2	1	3	1	PRPF3
OG0001874	1	1	1	1	5	1	RPS16
OG0002387	1	2	2	1	2	1	RPL36A
OG0002790	1	1	1	1	2	1	RPS27, RPS27L
OG0003812	1	1	1	1	3	1	RPS17
OG0004209	1	1	2	1	2	1	MRPL34
OG0004660	1	1	1	1	2	1	RPS7
OG0004684	1	1	1	1	2	1	RPS3A
OG0005184	1	1	1	1	2	1	MRPS5
OG0005583	1	1	1	1	2	1	RPL5
OG0005688	1	1	1	1	2	1	OSCP1
OG0006153	1	1	1	1	2	1	MRPL11
OG0006422	1	1	1	1	2	1	RPS21

Orthogroups and the number of genes in each species and *A. homolechis* gene names that comprise each orthogroup with the GO term “translation,” where gene numbers were estimated to expand in the *A. homolechis* terminal branch

### Estimation of the DNA substitution rate and divergence time

DNA substitution rates (substitutions per year) at fourfold degenerate (4D) sites for the branches of the *Anolis* lizards clade in the phylogeny, estimated using a Bayesian method implemented in MCMCTree, ranged from 1.13 × 10^−9^ to 2.20 × 10^−9^ (Additional file 1: Fig. S4), with an average of 1.8 × 10^−9^. The age of the common ancestor of *Anolis* lizards included in this study and the divergence times of Cuban *Anolis* lizards were estimated to be 51.7 million years ago (mya) in the Eocene and 7.71–38.5 mya from the Eocene to the Miocene, respectively (Additional file 1: Fig. S5).

### Estimation of population size history

**Fig. 5:**
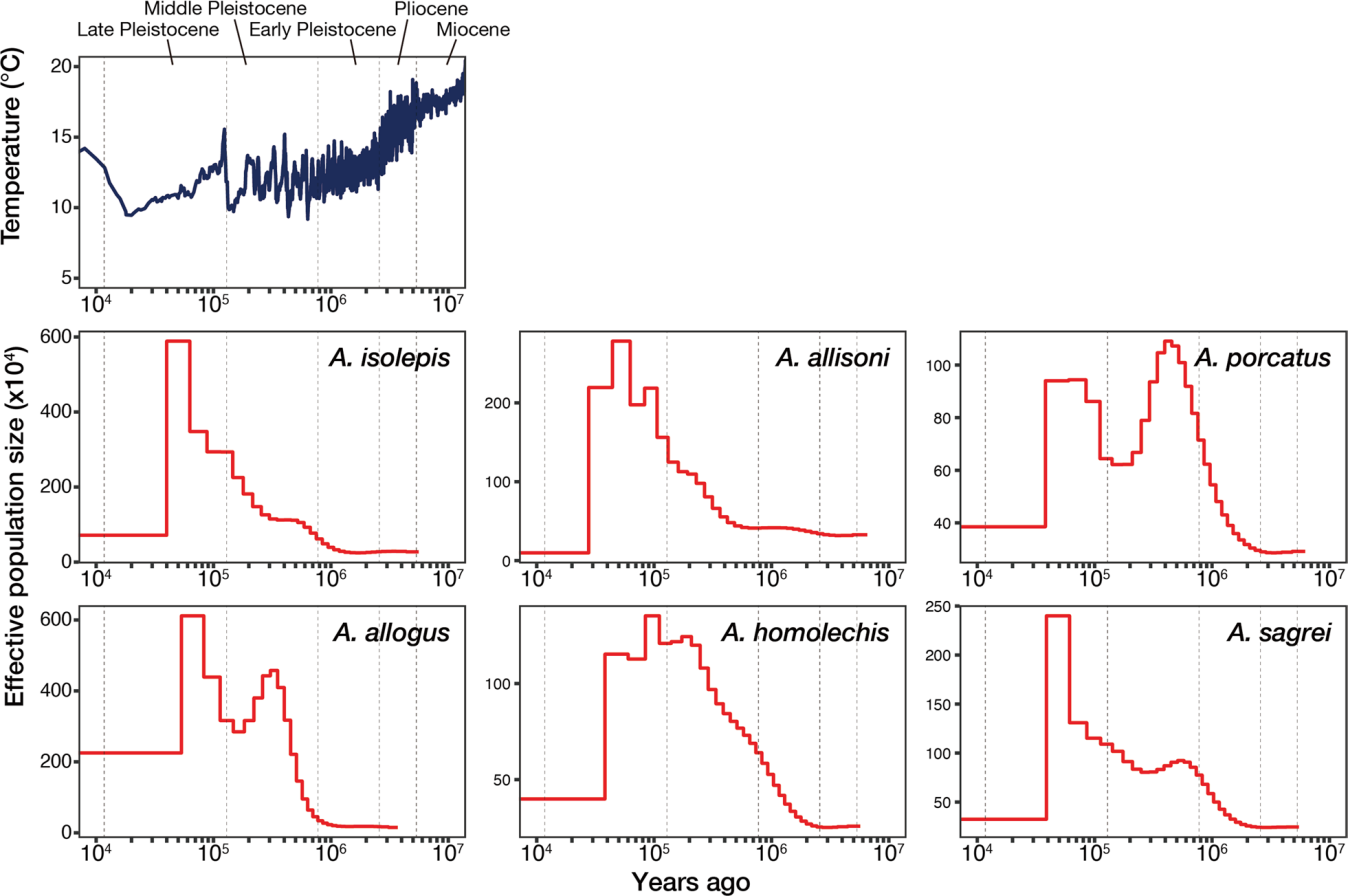
Past effective population sizes of the six Cuban *Anolis* lizards. Top: the Earth’s surface temperature [36]; six lower panels: the estimated past effective population sizes of six Cuban *Anolis* lizards estimated using the Pairwise Sequentially Markovian Coalescent (PSMC) model. The generation time was set as one year for all six species. The mutation rates for *A. isolepis*, *A. allisoni*, *A. porcatus*, *A. allogus*, *A. homolechis*, and *A. sagrei* were set as 1.4 × 10^−9^, 1.5 × 10^−9^, 1.8 × 10^−9^, 1.6 × 10^−9^, 2.0 × 10^−9^, and 2.1 × 10^−9^, respectively

To estimate the population size history of the six Cuban *Anolis* lizards (*A. isolepis*, *A. allisoni*, *A. porcatus*, *A. allogus*, *A. homolechis*, and *A. sagrei*), we estimated the past effective population size of each species using Pairwise Sequentially Markovian Coalescent (PSMC) based on mapping back the sequence reads to the genome assemblies and the heterozygous site calling results. Assuming a generation time of one year and a mutation rate of *A. isolepis*, *A. allisoni*, *A. porcatus*, *A. allogus*, *A. homolechis*, and *A. sagrei* of 1.4 × 10^−9^, 1.5 × 10^−9^, 1.8 × 10^−9^, 1.6 × 10^−9^, 2.0 × 10^−9^, and 2.1 × 10^−9^, respectively, the history of effective population sizes since the Pliocene or the latest Miocene, when the divergence of the species in this study was estimated to have ended, was calculated for each species (Fig. 5; Additional file 1: Fig. S6). An increase in the effective population size was estimated for all six species. Furthermore, a significant drop in the effective population size was estimated to have occurred in the Middle Pleistocene for *A. porcatus*, *A. allogus*, and *A. sagrei*. For *A. isolepis*, there was a stagnation in the increase in effective population size during this period. However, for *A. allisoni* and *A. homolechis*, these changes were less pronounced or later than for other species. Change processes in the effective population sizes of these species did not appear to be linked to the global temperature [36] (Fig. 5).

## Discussion

We report the genomes of six Cuban *Anolis* lizards (*A. isolepis*, *A. allisoni*, *A. porcatus*, *A. allogus*, *A. homolechis*, and *A. sagrei*), three each from trunk-crown (*A. isolepis*, *A. allisoni*, and *A. porcatus*) and trunk-ground (*A. allogus*, *A. homolechis*, and *A. sagrei*) lineages of Cuban *Anolis* lizards, which also have varied habitats. These genome assemblies provide genomic resources for elucidating the genetic basis of diversification and adaptive evolution. In this study, using comparative analyses of these genomes with those of mainland *Anolis* lizards reported in previous studies, we attempted to elucidate the origin and accumulation process of genetic variation during the diversification of *Anolis* lizards.

The completed BUSCO coverage was high (77.5–86.9%) in the six genome assemblies reconstructed in this study, comparable to that of the *A. carolinensis* genome (AnoCar2.0) (88.4%), which was assembled to the chromosome level (Table 2, Fig. 1). Furthermore, the number of genes predicted for each species was 21,688–25,839 (Table 3), similar to the number deposited in Ensembl release 104 (21,555) for *A. carolinensis*. Thus, our novel draft genomes have high gene coverage.

The GC content appeared to be slightly higher in the three Cuban trunk-ground *Anolis* lizards than in the three Cuban trunk-crown *Anolis* lizards and their relative, *A. carolinensis* (Fig. 2). Furthermore, the shape of the GC content distribution differed between the two mainland species (*A. frenatus* and *A. auratus*) and the six Cuban species in addition to *A. carolinensis* (Fig. 2). However, when GC distributions were created only for regions other than those detected as repeat elements, there were no significant differences between the Cuban trunk-crown species and trunk-ground species and between *A. auratus* and other species in the GC content distribution (Additional file 1: Fig. S1), suggesting that differences may be due to variations in the composition of repeat elements in these genomes. Moreover, the assembly sizes of the six Cuban *Anolis* lizard draft genomes were smaller than their estimated genome sizes and still had many gaps (5.6–9.3% of the genomes). Previously, Costantini et al. (2016) [37] argued that the *A. carolinensis* genome had many gaps due to its high GC content, making sequencing difficult. Therefore, the actual difference in GC content and distribution among lineages or species remains uncertain. A comparison of the GC landscape at the chromosome level of genome assemblies with few gaps is still required.

The average estimated DNA substitution rate (substitutions per year) for *Anolis* lizard branches was 1.8 × 10^˗9^, close to that calculated for mainland *Anolis* lizards by Tollis et al. (2018) [21]. The estimated age of the common ancestor of *Anolis* lizards included in this study, 51.7 mya (Additional file 1: Fig. S4), was roughly consistent with that reported in previous studies [20, 38, 39]. The estimated divergence times of Cuban *Anolis* lizards, 7.71–38.5 mya (Additional file 1: Fig. S4), are also roughly consistent with previous estimations [38, 39]. However, the divergence time of Cuban *Anolis* lizards is slightly more recent than previous estimates. Although considerably more genes and sites were used in our analysis than in previous studies, functional constraints were observed in 4D sites [40], which were used for the analysis. Therefore, the results of this study may also be biased.

We observed some differences in the composition of repeat elements among species. By comparing the composition of repeat elements and the repeat landscape for six Cuban *Anolis* lizards plus three mainland *Anolis* lizards (*A. carolinensis*, *A. auratus*, and *A. apletophallus*) with considering phylogenetic relationships and the divergence time of these species, we developed an overview of the repeat element accumulation process during the diversification of *Anolis* lizards. Bourgeois et al. [41] showed that the genome of *A. carolinensis* contains many Gypsy family LTRs compared to its Cuban relatives. We also observed this in the present study (Fig. 3; Additional file 1: Tables S3–S11), and the accumulation of LTRs is estimated to have peaked at approximately 10 mya (Fig. 3). Since the divergence times between *A. carolinensis* and *A. allisoni* as well as *A. carolinensis* and *A. isolepis* were estimated as 7.71 and 14.03 mya, respectively, the LTRs amplified in the common ancestor of *A. carolinensis* and *A. allisoni* might have accumulated in the ancestors of *A. carolinensis*. Additionally, looking at the overall shape of the repeat landscapes, those of the trunk-crown Cuban *Anolis* lizards and *A. carolinensis* had more recent peaks than those of other species. Although the shape was steeper in trunk-ground Cuban *Anolis* lizards, the peak age for the closely related *A. auratus* and *A. apletophallus* was similar to those of trunk-ground Cuban *Anolis* lizards. These results show that each lineage underwent different accumulation processes of repeat elements. These results also indicate that the genomes of three Cuban *Anolis* lizards contained more LINEs than other *Anolis* lizards (Fig. 3; Additional file 1: Tables S3–S11). However, by comparing their breakdown (Additional file 1: Tables S3–S11) and repeat landscapes (Additional file 1: Fig. S3), differences were possible in the accumulation processes of several LINE families among these species. Therefore, their common ancestor may have experienced alterations in common transposition mechanisms among the LINE families. Alternatively, each family of LINEs may have accumulated due to genetic drift in each species.

Significant differences in the ratio of duplicated genes to single-copy genes were detected among Cuban *Anolis* lizards in both the trunk-crown and trunk-ground lineages. The results showed that the forest species *A. isolepis* and *A. allogus* had a lower *P*_D_ than open area and forest margin species in each lineage. Notably, *A. isolepis*, which lives only in mountainous cloud forests or rainforests > 800 m above sea level [11], had the lowest *P*_D_. Significant differences were also detected in the ratio of duplicated genes to single-copy genes between *A. porcatus* and *A. allisoni*, which inhabit open areas in the trunk-crown lineage, and between *A. homolechis* and *A. sagrei*, which inhabit the forest margin and open areas, respectively, in the trunk-ground lineage. *A. homolechis*, which had the highest *P*_D_, have a wider range of body temperatures in natural areas in both males and females than other species [10, 12]. Gene duplication is a potent source of the variation required for adaptive evolution. Makino and Kawata (2012) [42] and Tamate et al. (2014) [43] reported that the range of habitat climates is positively correlated with *P*_D_*.* Forests can buffer temperature under canopis [44, 45]. Therefore, it is conceivable that the range of climatic conditions was narrower in forests than in open areas and at forest edges. This may be related to the *P*_D_ of the Cuban *Anolis* lizards. Analysis of gene family expansion or contraction indicates that numerous expansions in the number of genes encoding ribosomal proteins occurred in *A. homolechis*. Akashi et al. [30] showed that in *A. homolechis,* significantly more genes encoding ribosomal proteins were highly expressed under heat-acclimated condition compared with *A. allogus* and *A. sagrei*. Thus, the relationship between the expansion of genes encoding ribosomal proteins and the evolution of gene expression regulation during heat acclimation should to be examined further. Makino and Kawata (2019) [46] also indicated that invasive species have high *P*_D_*. A. porcatus* is a close relative of *A. carolinensis*, which is native to the United States and has invaded many parts of the world. The higher *P*_D_ in *A. porcatus* than in *A. allisoni* may be related to the invasiveness of *A. porcatus.* Given that the evolution of new gene copies by duplication may facilitate advances into novel environments, it is also possible that the direction of evolution, i.e., whether the habitat is ancestral or not, is related to *P*_D_. Thus, to confirm the relationship between *P*_D_ and natural habitats, examining the habitat transition process and analyzing *P*_D_ in more species is necessary. However, while gene duplication may promote adaptive evolution, the retention of duplicated genes is associated with a fitness cost [47]. Since *A. homolechis* and *A. porcatus* had smaller maximum effective population sizes than other species of trunk-ground and trunk-crown lineages, respectively, during the period in which the estimation was done, new deleterious gene copies may be less likely to be removed than in other species, impacting the high *P*_D_ of these two species. The relationship between the gene duplications detected in this study and adaptive evolution should be investigated in the future.

Estimating population size history can provide critical information for examining the evolutionary process of organisms. We estimated the past effective population sizes of six Cuban *Anolis* lizards since the Pliocene or the latest Miocene (Fig. 5). If the mutation rate and generation time set in this study are correct, the following can be inferred: Although an increase in effective population size was estimated for all six species, in *A. isolepis*, *A. porcatus*, *A. allogus*, and *A. sagrei*, this ceased in the middle Pleistocene. However, for *A. allisoni* and *A. homolechis*, such changes were less pronounced or appeared later than in the other species. Paleogeographic studies suggest that, although high altitudes in parts of central and eastern Cuba were not inundated under the sea, many parts of Cuba were near the boundary between the middle and late Pleistocene compared to the Pliocene and early Pleistocene periods and the present-day [48, 49]. Individuals of *A. isolepis*, *A. allogus*, and *A. sagrei*, whose genomes were analyzed in this study, were collected from western Cuba. Therefore, the fluctuation in habitat range due to inundation under the sea may have contributed to the population size fluctuation of these species in western Cuba. The smaller fluctuation for *A. isolepis* compared to *A. allogus* and *A. sagrei* may be because the distribution of *A. isolepis* was limited to high-elevation mountainous areas [11]. An individual of *A. homolechis* was collected from eastern Cuba. The effect of fluctuations in sea level on population size was assumed to be smaller or have occurred later in eastern Cuba than in western Cuba. *A. porcatus* and *A. allisoni* samples were collected in the central part of the country, but for *A. porcatus*, severe fluctuations in the effective population size were inferred. *A. porcatus* and *A. allisoni* are closely related, with the larger-sized *A. allisoni* currently dominating central Cuba [11, 50]. Since the beginning of the decline in the effective population size of *A. porcatus* and the increase in the effective population size of *A. allisoni* roughly overlapped in the middle Pleistocene, the pattern of fluctuations in the effective population sizes of these populations in central Cuba may reflect intense interspecies competition. Moreover, an increase in the effective population size of *A. isolepis* and *A. allogus*, which currently inhabit forests, was estimated at around 1 mya. In contrast, that of *A. porcatus*, *A. sagrei*, and *A. homolechis*, which currently inhabit open areas or forest margins, was estimated a little earlier, around 2 mya. How long the current habitat of each species has remained the same is unknown, but if it has been consistent since 2 mya, such differences may be due to differences in habitats. Thus, if the time scale of the estimation is correct, it is conceivable that the history of the population sizes of these six Cuban *Anolis* lizards is related to Cuba’s geohistory, interspecies competition, and habitats.

In this study, we reconstructed novel genome assemblies of six Cuban *Anolis* lizards with relatively long scaffolds and high gene completeness. By examining and comparing each genome feature, including those of mainland species reported in previous studies, we estimated the genetic variation that occurred during the diversification of *Anolis* lizards. Interspecies comparisons of the TE and gene composition revealed different TE accumulation processes and gene copy number changes for each species or lineage. Furthermore, estimates of the past effective population size suggest that the population sizes of these species may have fluctuated due to geohistory or interspecies competition. These results provide essential information as a stepping stone for elucidating the diversification and adaptive radiation mechanism of *Anolis* lizards. Additional improvements in the quality of the novel genome assemblies reconstructed in this study are expected to advance population and epigenome analyses at the genome level.

## Conclusions

In this study, we reconstructed novel genome assemblies of six Cuban *Anolis* lizards with relatively long scaffolds and high gene completeness. We performed comparative analyses of these genome assemblies to investigate genetic changes that occurred during their diversification. Repetitive element analysis showed species and lineage-specific transposon accumulation processes. Additionally, we evaluated gene copy number evolution, considered to be associated with habitat adaptation. Furthermore, estimates of past effective population sizes suggest that the population size of Cuban *Anolis* lizards may have fluctuated due to Cuba’s geohistory and interspecies competition. These results provide novel insights into the genetic changes occurring during the diversification of *Anolis* lizards and will allow additional investigations into their diversification mechanisms.

## Materials and methods

### Sample preparation, genome sequencing, and de novo assembly

Specimens of *Anolis* species were collected from Cuba: individuals for *A. isolepis*, *A. allogus*, and *A. sagrei* were sampled from Las Terrazas, Artemisa, *A. porcatus* from Topes de Collantes, Sancti Spíritus, *A. homolechis* from Macambo, Guantánamo, and *A. allisoni* from Trinidad, Sancti Spíritus. The samples were collected from 2012 to 2018.

Sample collection in Cuba, exportation to Japan, and use for research were approved by the Centro de Control y Gestión Ambiental of the Agencia de Medio Ambiente de Cuba. One individual of each species was collected. The *A. isolepis*, *A. porcatus*, and *A. sagrei* individuals were females, and those of *A. allisoni*, *A. allogus*, and *A. homolechis* were males. The animals were euthanized by decapitation, followed by the extraction of brain tissues (the method of euthanasia was approved by the Committee on Animal Experiments of Tohoku University, Permit number: 2012LsA-019, 2013LsA-023, 2016LsA-011). Then, the brain and muscle tissues were preserved in 99% ethanol or RNA*later*^®^ tissue storage reagent (Ambion, Foster City, CA, USA) and stored at − 15 °C to − 80 °C until DNA extraction. DNA was extracted from the brain for *A. isolepis*, *A. porcatus*, *A. allisoni*, and *A. homolechis* and the muscle for *A. sagrei* and *A. allogus* according to the HMW gDNA Extraction Protocol in the Sample Preparation Demonstrated Protocol provided by 10 × Genomics. Library preparation was performed using Chromium Controller (10 × Genomics, Pleasanton, CA, USA) according to the Genome Reagent Kit v.2 User Guide provided by 10 × Genomics, after which the genome libraries were sequenced with an Illumina HiSeq X (2 × 150-bp reads). The yielded reads for each genome were de novo assembled using Supernova v.2.1.1 [35] with an ideal coverage of ~ 56 × [35]. First, we conducted the assembly with all the yielded reads. Then, if Supernova reported that the coverage was > 56 × , we calculated the appropriate number of reads that would bring the coverage to about 56 × using the estimated genome size, and then, conducted the assembly again by setting this number of reads. Haplotigs and heterozygous overlapping duplications were removed as follows: First, to obtain cleaned sequence reads, 10 × barcodes in the sequence reads were removed using Longranger basic (https://support.10xgenomics.com/genome-exome/software/pipelines/latest/what-is-long-ranger), after which the NGS QC Toolkit [51] was used to perform quality filtering of the reads. The clean reads were then mapped back to each assembled genome using BWA [52] and Minimap2 [53]. Haplotigs and overlaps in the genomes were detected and removed using purge_dups [54] based on the depth of short-read alignments. The gene completeness of the genome assemblies before and after the removal of haplotigs and overlaps was assessed by examining the completeness of the BUSCOs for vertebrates (odb9) using BUSCO software [55]. As shown in the results section, the percentage of duplicated vertebrate BUSCOs decreased after removing the haplotigs and overlaps. Therefore, those with haplotigs and overlaps removed were considered the final assemblies for subsequent analyses.

### Annotation of repeat elements and drawing repeat landscapes

To compare the composition of repeat elements in the genomes and survey the repeat element accumulation process, we detected repeat elements in the genome assemblies. We estimated the historical dynamics of repeat element accumulation in the following steps: First, repeat elements in all *Anolis* genomes included in this study were de novo searched using RepeatModeler [56]. Next, the resulting library of de novo repeat elements was integrated with the *Anolis* repeat sequence library already deposited in Repbase [57]. The integrated library was then used to detect repeat elements in the genomes and categorize them into classes and families using RepeatMasker [58]. Afterward, the detected repeat elements were aligned to consensus sequences for each family by RepeatMasker, and Kimura’s two-parameter distances (K2P distances) were calculated using calcDivergenceFromAlign.pl in the RepeatMasker package. Subsequently, a histogram based on the calculated K2P distances (the repeat landscape) was plotted for each genome using createRepeatLandscape.pl from the RepeatMasker package to estimate the historical dynamics of repeat element accumulation, and we converted the K2P distance of the repeat landscape into years using parseRM.pl (available at https://github.com/4ureliek/Parsing-RepeatMasker-Outputs, [59]). We used the average DNA substitution rates estimated for *Anolis* lizard branches for each species repeat landscape conversion as described in the “Estimation of DNA substitution rate and divergence time” section.

Among the available genome assemblies of three *Anolis* lizards (*A. frenatus, A. auratus*, and *A. apletophallus*) [21], the *A. frenatus* genome was excluded because the assembly quality was not suitable for repeat element analysis. The *A. frenatus* genome has 25% and 20% G and C abundance, respectively, which significantly deviate from Chargaff’s rule (A ≈ T and G ≈ C) that can be used to evaluate the assembly quality [60, 61].

### Gene annotation, ortholog grouping, and duplicated gene identification

Gene models for the repeat-masked genome assemblies of *A. isolepis*, *A. allisoni*, *A. porcatus*, *A. allogus*, *A. homolechis*, and *A. sagrei* were constructed using BRAKER v.2.1.6 [62] with RNA-seq data and protein sequences of *A. carolinensis* obtained from Ensemble release 104. RNA-seq reads of each *Anolis* lizard [30, 32], available from the DDBJ Sequence Read Archive (DRA) of the DNA Data Bank of Japan (DDBJ) (Additional file 1: Table S12), were aligned to each genome assembly with GSNAP [63]. Other nondefault options in BRAKER were –etpmode, –softmasking, and –gff3. A BLAST search was used to identify predicted protein-coding genes that did not match any protein sequences in the UniProtKB/Swiss-Prot database. Funannotate v.1.8.7 [64] was used to filter out these genes, predict UTR regions, and annotate gene names and GO terms for each gene.

To identify duplicated genes, predicted genes were clustered into orthogroups using OrthoFinder v.2.5.4 [65, 66] using the longest protein sequences of genes predicted from the genomes of *A. isolepis*, *A. allisoni*, *A. porcatus*, *A. allogus*, *A. homolechis*, *A. sagrei*, and *Pogona vitticeps*, which was selected as the outgroup. The sequences of *P. vitticeps* were retrieved from Ensembl release 104. When multiple proteins from a single species were grouped into the same orthogroup, genes encoding these proteins were considered duplicated. We then calculated *P*_D_ for each species. We assumed that differences in annotation methods and genome assembly quality would make comparing gene numbers and *P*_D_ difficult. Therefore we included only six Cuban *Anolis* lizards, for which genome assembly and annotation were conducted in this study, for comparative analysis of duplicate genes. The independence of the ratio of duplicated to single gene numbers and species was tested separately for three closely related Cuban trunk-crown *Anolis* lizards (*A. isolepis*, *A. allisoni*, and *A. porcatus*) and three closely related Cuban trunk-ground *Anolis* lizards (*A. allogus*, *A. homolechis*, and *A. sagrei*) by G-test with STP [67] to see whether there were significant differences in *P*_D_ among species, and if so, which species had significant differences in *P*_D_.

The expansion or contraction of the number of genes was estimated using CAFE v.4.2.1 [68] for orthogroups comprising genes from all species. Before the estimation, the genome assemblies and annotation errors were estimated using “caferror.py,” included in the CAFE package. The ultrametric input tree was prepared according to the following procedure: First, a maximum likelihood (ML) tree was created with RAxML [69] using 4D sites shared by > 80% of the species extracted from the alignment results of the coding sequences of single-copy orthogroups using PRANK [70]. Then, the ML tree was converted to an ultrametric tree using r8s [71] with the divergence time (146 mya) of *Anolis* lizards and the outgroup *Pogona vitticeps* referenced from TimeTree [72].

### Estimation of DNA substitution rate and divergence time

We used the 4D sites of single-copy ortholog genes shared by 41 species of sarcopterygian vertebrate species to reconstruct phylogenetic trees and estimate the DNA substitution rates and divergence times of *Anolis* species. These 41 sarcopterygian vertebrates include 34 species selected from the major groups of amniotes (mammals, birds, crocodilians, turtles, and squamates), the six species of Cuban *Anolis* lizards in this study, and a coelacanth as the outgroup (Additional file 1: Table S13). The protein sequences from these species were obtained from the databases listed in Additional file 1: Table S13. The coding sequences of *Gekko japonicus*, *A. apletophallus*, *A. auratus*, and *A. frenatus* were extracted from their respective genome assemblies referring to annotation data using GffRead [73]. Databases from which the coding sequences of other species were obtained are also listed in Additional file 1: Table S13. A total of 41 species were analyzed, including the six Cuban *Anolis* lizards for which genome assemblies were reconstructed in this study. Their single-copy orthologs were grouped using SonicParanoid [74], comparing the longest protein sequences for each gene. The coding sequences of single-copy orthologs were aligned for each orthogroup with the codon model using PRANK [70], and 4D sites shared by 80% of these species were extracted from the alignment. We estimated the DNA substitution rate and divergence time with the Bayesian relaxed molecular clock approach using MCMCTree in the PAML package (version 4.9j) [75]. In advance, the substitution rate was roughly estimated using baseml in the PAML package (version 4.8) [75], with a fixed calibration point for the root of 418 mya [76] to estimate a prior distribution of the substitution rate. Then, MCMCTree sampled 10,000 times, with a sampling frequency of 10,000, after a burn-in of 25,000,000 iterations. An ML tree was reconstructed using RAxML [69] with the GTR + Γ model. We used this ML tree without branch lengths as the input tree. Calibration points were set as previously described by Benton et al. (2015) [76] as follows: 418 mya for the fixed root age; 256–296 mya for Diapsida; 247–260 mya for Archosauria; 169–210 mya for Squamata; 165–202 mya for Mammalia; and 66–87 mya for Neognathae. The average DNA substitution rates of the *Anolis* lizard branches and each rate for these species’ terminal branches obtained from this analysis were used to estimate the time scale of the accumulation processes of TEs and the effective population size history, respectively.

### Estimation of past effective population sizes

The past effective population sizes of the six Cuban *Anolis* lizards were estimated using the PSMC model [77]. After removing the 10 × barcodes and conducting quality filtering, the sequence reads were mapped back to the corresponding genome assemblies of each species using BWA [52]. SNPs were called in regions where reads were mapped at a depth of one-third to twice the average genome depth using samtools and bcftools [78], following the method recommended by the README of the PSMC software. The input consensus sequence was generated using bcftools [78] for each genome. PSMC analyses were run with a time interval pattern and maximum 2N_0_ coalescent time set to “4 + 30 × 2 + 4 + 6 + 10” and 5, respectively, with 100 bootstrap replicates. When rescaling the time and population size, the generation time was set to one year and the mutation rate to 1.4 × 10^−9^, 1.5 × 10^−9^, 1.8 × 10^−9^, 1.6 × 10^−9^, 2.0 × 10^−9^, and 2.1 × 10^−9^ for *A. isolepis*, *A. allisoni*, *A. porcatus*, *A. allogus*, *A. homolechis*, and *A. sagrei*, respectively, which were estimated from the phylogenetic analysis.

## Supplementary Information

**Additional file 1.** Additional tables and figures.

## Data Availability

The paired-end reads used for de novo genome assembly were deposited in the DDBJ Short Read Archive database (https://www.ddbj.nig.ac.jp/dra/index-e.html) under accession number DRA013941. The set of the 10 × barcode fastq files, soft-masked genome assemblies, gene models, and predicted coding nucleotide and peptide sequences are available at figshare (https://figshare.com/projects/Reconstruction_of_draft_genomes_for_six_Cuban_Anolis_lizards/137421).
