# Supplementary material for: Draft genome of six Cuban Anolis lizards and insights into genetic changes during their diversification
Source: BMC Ecol Evol. 2022 Nov 4;22:129. doi: 10.1186/s12862-022-02086-7 (PMC9635203; doi:10.1186/s12862-022-02086-7)
Supplement: Supplementary file 1 — Additional file 1. Additional tables and figures. [file 12862_2022_2086_MOESM1_ESM.docx]

**Additional Table 1. Genome sequencing and *de novo* genome assembly results before removing haplotigs and overlaps.**

| **Species** | **Total read data before adjusting**  **the number of reads**  **(Gb)** | **Coverage**  **before adjusting**  **the number of reads** | **Coverage**  **after adjusting**  **the number of reads** | **Contig N50**  **(Kb)** | **Scaffold N50**  **(Mb)** | **Total length**  **(Gb)** | **Number**  **≧ 10Kb** |
| --- | --- | --- | --- | --- | --- | --- | --- |
| *Anolis isolepis* | 128 | 61.70× | 56.64× | 62.59 | 28.69 | 1.80 | 3.36 K |
| *Anolis allisoni* | 129 | 57.34× | 56.27× | 43.31 | 5.69 | 1.94 | 7.31 K |
| *Anolis porcatus* | 134 | 63.30× | 56.80× | 49.41 | 22.02 | 1.95 | 7.90 K |
| *Anolis allogus* | 125 | 54.16× | ­ | 46.44 | 42.71 | 2.31 | 6.61 K |
| *Anolis homolechis* | 125 | 47.26× |  | 44.47 | 41.02 | 2.23 | 11.46 K |
| *Anolis sagrei* | 123 | 60.18× | 56.48× | 49.79 | 34.06 | 2.19 | 11.18 K |

**Additional Table 2. The vertebrate BUSCO (odb9) metrics of genome assemblies before and after purge of haplotigs and overlaps.** BUSCO: Benchmarking Universal Single-Copy Orthologs

|  | **Before purge of haplotigs and overlaps** | | | | **After purge of haplotigs and overlaps** | | | |
| --- | --- | --- | --- | --- | --- | --- | --- | --- |
| **Species** | **Complete** | **(Single** | **Duplicated)** | **Fragmented** | **Complete** | **(Single** | **Duplicated)** | **Fragmented** |
| *Anolis isolepis* | 88.0% | 86.2% | 1.8% | 7.7% | 86.9% | 86.1% | 0.8% | 7.0% |
| *Anolis allisoni* | 85.9% | 84.2% | 1.7% | 9.6% | 83.8% | 83.1% | 0.7% | 9.4% |
| *Anolis porcatus* | 85.7% | 83.4% | 2.3% | 10.0% | 83.7% | 83.1% | 0.6% | 9.4% |
| *Anolis allogus* | 86.7% | 82.4% | 4.3% | 8.5% | 86.0% | 83.8% | 2.2% | 8.4% |
| *Anolis homolechis* | 79.4% | 77.8% | 1.6% | 12.8% | 77.5% | 76.6% | 0.9% | 12.3% |
| *Anolis sagrei* | 86.0% | 83.6% | 2.4% | 9.4% | 85.0% | 84.0% | 1.0% | 8.9% |

**Additional Table 3. Content of repeat elements in the *Anolis isolepis* genome.**

|  | **Number of elements** | **Length occupied (bp)** | **Percentage of sequence** |
| --- | --- | --- | --- |
| **Retroelements** | 1878168 | 430006788 | 25.81 |
| **SINEs:** | 368401 | 60380616 | 3.62 |
| **Penelope** | 324880 | 45110106 | 2.71 |
| **LINEs:** | 1430507 | 333966228 | 20.04 |
| **CRE/SLACS** | 0 | 0 | 0 |
| **L2/CR1/Rex** | 770129 | 166127355 | 9.97 |
| **R1/LOA/Jockey** | 0 | 0 | 0 |
| **R2/R4/NeSL** | 38810 | 13264806 | 0.8 |
| **RTE/Bov-B** | 191555 | 56599760 | 3.4 |
| **L1/CIN4** | 61011 | 35495653 | 2.13 |
| **LTR elements:** | 79260 | 35659944 | 2.14 |
| **BEL/Pao** | 1374 | 861407 | 0.05 |
| **Ty1/Copia** | 25897 | 5566259 | 0.33 |
| **Gypsy/DIRS1** | 34004 | 22064860 | 1.32 |
| **Retroviral** | 11690 | 4262542 | 0.26 |
| **DNA transposons** | 815039 | 125105108 | 7.51 |
| **hobo-Activator** | 304052 | 42543725 | 2.55 |
| **Tc1-IS630-Pogo** | 341448 | 61163483 | 3.67 |
| **En-Spm** | 0 | 0 | 0 |
| **MuDR-IS905** | 0 | 0 | 0 |
| **PiggyBac** | 0 | 0 | 0 |
| **Tourist/Harbinger** | 124257 | 15460963 | 0.93 |
| **Other** | 0 | 0 | 0 |
| **Rolling-circles** | 103984 | 27359258 | 1.64 |
| **Unclassified:** | 857 | 132814 | 0.01 |
| **Total interspersed repeats:** |  | 555244710 | 33.32 |
| **Small RNA:** | 3962 | 421256 | 0.03 |
| **Satellites:** | 4873 | 1133691 | 0.07 |
| **Simple repeats:** | 464709 | 19405729 | 1.16 |
| **Low complexity:** | 42891 | 2546122 | 0.15 |

**Additional Table 4. Content of repeat elements in the *Anolis allisoni* genome.**

|  | **Number of elements** | **Length occupied (bp)** | **Percentage of sequence** |
| --- | --- | --- | --- |
| **Retroelements** | 1998283 | 472471996 | 27.06 |
| **SINEs:** | 493079 | 87160708 | 4.99 |
| **Penelope** | 200757 | 36629163 | 2.1 |
| **LINEs:** | 1416121 | 344051638 | 19.71 |
| **CRE/SLACS** | 0 | 0 | 0 |
| **L2/CR1/Rex** | 808487 | 177785673 | 10.18 |
| **R1/LOA/Jockey** | 0 | 0 | 0 |
| **R2/R4/NeSL** | 96899 | 33309694 | 1.91 |
| **RTE/Bov-B** | 200522 | 50301566 | 2.88 |
| **L1/CIN4** | 71273 | 33018779 | 1.89 |
| **LTR elements:** | 89083 | 41259650 | 2.36 |
| **BEL/Pao** | 2511 | 1608912 | 0.09 |
| **Ty1/Copia** | 13499 | 5445708 | 0.31 |
| **Gypsy/DIRS1** | 61792 | 28108497 | 1.61 |
| **Retroviral** | 7992 | 4342287 | 0.25 |
| **DNA transposons** | 935292 | 141605863 | 8.11 |
| **hobo-Activator** | 392108 | 59692810 | 3.42 |
| **Tc1-IS630-Pogo** | 349042 | 57717916 | 3.31 |
| **En-Spm** | 0 | 0 | 0 |
| **MuDR-IS905** | 0 | 0 | 0 |
| **PiggyBac** | 362 | 93855 | 0.01 |
| **Tourist/Harbinger** | 123305 | 14740173 | 0.84 |
| **Other** | 0 | 0 | 0 |
| **Rolling-circles** | 90446 | 15765657 | 0.9 |
| **Unclassified:** | 847 | 130180 | 0.01 |
| **Total interspersed repeats:** | na | 614208039 | 35.18 |
| **Small RNA:** | 29050 | 3533512 | 0.2 |
| **Satellites:** | 5810 | 1242447 | 0.07 |
| **Simple repeats:** | 523272 | 21575630 | 1.24 |
| **Low complexity:** | 43639 | 2563403 | 0.15 |

**Additional Table 5. Content of repeat elements in the *Anolis porcatus* genome.**

|  | **Number of elements** | **Length occupied (bp)** | **Percentage of sequence** |
| --- | --- | --- | --- |
| **Retroelements** | 1930718 | 436494158 | 25.05 |
| **SINEs:** | 461468 | 83754369 | 4.81 |
| **Penelope** | 246114 | 39263710 | 2.25 |
| **LINEs:** | 1365220 | 314491428 | 18.05 |
| **CRE/SLACS** | 0 | 0 | 0 |
| **L2/CR1/Rex** | 647860 | 142289738 | 8.17 |
| **R1/LOA/Jockey** | 0 | 0 | 0 |
| **R2/R4/NeSL** | 82081 | 26647143 | 1.53 |
| **RTE/Bov-B** | 184598 | 47877138 | 2.75 |
| **L1/CIN4** | 53402 | 27608041 | 1.58 |
| **LTR elements:** | 104030 | 38248361 | 2.2 |
| **BEL/Pao** | 2372 | 1477202 | 0.08 |
| **Ty1/Copia** | 13214 | 5295606 | 0.3 |
| **Gypsy/DIRS1** | 75415 | 26203429 | 1.5 |
| **Retroviral** | 5210 | 3173081 | 0.18 |
| **DNA transposons** | 1050505 | 160076517 | 9.19 |
| **hobo-Activator** | 572817 | 80050700 | 4.59 |
| **Tc1-IS630-Pogo** | 344059 | 63690746 | 3.66 |
| **En-Spm** | 0 | 0 | 0 |
| **MuDR-IS905** | 0 | 0 | 0 |
| **PiggyBac** | 331 | 91451 | 0.01 |
| **Tourist/Harbinger** | 95172 | 11036882 | 0.63 |
| **Other** | 0 | 0 | 0 |
| **Rolling-circles** | 91492 | 19842974 | 1.14 |
| **Unclassified:** | 854 | 130249 | 0.01 |
| **Total interspersed repeats:** | na | 596700924 | 34.24 |
| **Small RNA:** | 3977 | 314281 | 0.02 |
| **Satellites:** | 8895 | 1547840 | 0.09 |
| **Simple repeats:** | 541400 | 23108516 | 1.33 |
| **Low complexity:** | 44267 | 2558821 | 0.15 |

**Additional Table 6. Content of repeat elements in the *Anolis allogus* genome.**

|  | **Number of elements** | **Length occupied (bp)** | **Percentage of sequence** |
| --- | --- | --- | --- |
| **Retroelements** | 2662359 | 642619412 | 30.72 |
| **SINEs:** | 318314 | 42786880 | 2.05 |
| **Penelope** | 176743 | 37730635 | 1.8 |
| **LINEs:** | 2241071 | 561848287 | 26.86 |
| **CRE/SLACS** | 0 | 0 | 0 |
| **L2/CR1/Rex** | 1294566 | 298470694 | 14.27 |
| **R1/LOA/Jockey** | 1668 | 459265 | 0.02 |
| **R2/R4/NeSL** | 35650 | 12561606 | 0.6 |
| **RTE/Bov-B** | 303421 | 76898337 | 3.68 |
| **L1/CIN4** | 69816 | 39473943 | 1.89 |
| **LTR elements:** | 102974 | 37984245 | 1.82 |
| **BEL/Pao** | 28800 | 8259095 | 0.39 |
| **Ty1/Copia** | 18194 | 5559362 | 0.27 |
| **Gypsy/DIRS1** | 30908 | 17969621 | 0.86 |
| **Retroviral** | 16895 | 3008300 | 0.14 |
| **DNA transposons** | 903077 | 149896799 | 7.17 |
| **hobo-Activator** | 439389 | 77691128 | 3.71 |
| **Tc1-IS630-Pogo** | 283381 | 50504196 | 2.41 |
| **En-Spm** | 0 | 0 | 0 |
| **MuDR-IS905** | 0 | 0 | 0 |
| **PiggyBac** | 282 | 62278 | 0 |
| **Tourist/Harbinger** | 129608 | 15453741 | 0.74 |
| **Other** | 0 | 0 | 0 |
| **Rolling-circles** | 133030 | 35287662 | 1.69 |
| **Unclassified:** | 897 | 139208 | 0.01 |
| **Total interspersed repeats:** | na | 792655419 | 37.89 |
| **Small RNA:** | 9454 | 679118 | 0.03 |
| **Satellites:** | 1593 | 145840 | 0.01 |
| **Simple repeats:** | 630240 | 32035685 | 1.53 |
| **Low complexity:** | 63954 | 6340749 | 0.3 |

**Additional Table 7. Content of repeat elements in the *Anolis homolechis* genome.**

|  | **Number of elements** | **Length occupied (bp)** | **Percentage of sequence** |
| --- | --- | --- | --- |
| **Retroelements** | 2343675 | 597838716 | 30.37 |
| **SINEs:** | 305541 | 51252321 | 2.6 |
| **Penelope** | 215376 | 44338146 | 2.25 |
| **LINEs:** | 1971309 | 516421231 | 26.23 |
| **CRE/SLACS** | 0 | 0 | 0 |
| **L2/CR1/Rex** | 1158109 | 287403950 | 14.6 |
| **R1/LOA/Jockey** | 0 | 0 | 0 |
| **R2/R4/NeSL** | 53553 | 13242368 | 0.67 |
| **RTE/Bov-B** | 320262 | 84847075 | 4.31 |
| **L1/CIN4** | 52083 | 28870316 | 1.47 |
| **LTR elements:** | 66825 | 30165164 | 1.53 |
| **BEL/Pao** | 12206 | 7709023 | 0.39 |
| **Ty1/Copia** | 5374 | 2218471 | 0.11 |
| **Gypsy/DIRS1** | 33551 | 14359346 | 0.73 |
| **Retroviral** | 2864 | 1255456 | 0.06 |
| **DNA transposons** | 935915 | 135737454 | 6.89 |
| **hobo-Activator** | 513728 | 70880574 | 3.6 |
| **Tc1-IS630-Pogo** | 234336 | 42194176 | 2.14 |
| **En-Spm** | 0 | 0 | 0 |
| **MuDR-IS905** | 0 | 0 | 0 |
| **PiggyBac** | 203 | 47232 | 0 |
| **Tourist/Harbinger** | 138615 | 16355869 | 0.83 |
| **Other** | 0 | 0 | 0 |
| **Rolling-circles** | 147579 | 25177678 | 1.28 |
| **Unclassified:** | 871 | 135886 | 0.01 |
| **Total interspersed repeats:** | na | 733712056 | 37.27 |
| **Small RNA:** | 51342 | 5240052 | 0.27 |
| **Satellites:** | 2555 | 1038946 | 0.05 |
| **Simple repeats:** | 603318 | 27492589 | 1.4 |
| **Low complexity:** | 54762 | 4429680 | 0.23 |

**Additional Table 8. Content of repeat elements in the *Anolis sagrei* genome.**

|  | **Number of elements** | **Length occupied (bp)** | **Percentage of sequence** |
| --- | --- | --- | --- |
| **Retroelements** | 2522210 | 579096787 | 30.43 |
| **SINEs:** | 282067 | 40211670 | 2.11 |
| **Penelope** | 210517 | 40834967 | 2.15 |
| **LINEs:** | 2194773 | 519825794 | 27.31 |
| **CRE/SLACS** | 0 | 0 | 0 |
| **L2/CR1/Rex** | 1296452 | 301555406 | 15.85 |
| **R1/LOA/Jockey** | 0 | 0 | 0 |
| **R2/R4/NeSL** | 46306 | 12524841 | 0.66 |
| **RTE/Bov-B** | 330586 | 83497794 | 4.39 |
| **L1/CIN4** | 49161 | 22657559 | 1.19 |
| **LTR elements:** | 45370 | 19059323 | 1 |
| **BEL/Pao** | 5336 | 2436534 | 0.13 |
| **Ty1/Copia** | 7430 | 3242174 | 0.17 |
| **Gypsy/DIRS1** | 17925 | 9717670 | 0.51 |
| **Retroviral** | 8711 | 1035769 | 0.05 |
| **DNA transposons** | 927191 | 131317724 | 6.9 |
| **hobo-Activator** | 479381 | 66069988 | 3.47 |
| **Tc1-IS630-Pogo** | 272130 | 44239274 | 2.32 |
| **En-Spm** | 0 | 0 | 0 |
| **MuDR-IS905** | 0 | 0 | 0 |
| **PiggyBac** | 0 | 0 | 0 |
| **Tourist/Harbinger** | 134818 | 15560202 | 0.82 |
| **Other** | 0 | 0 | 0 |
| **Rolling-circles** | 160026 | 33123415 | 1.74 |
| **Unclassified:** | 882 | 137231 | 0.01 |
| **Total interspersed repeats:** | na | 710551742 | 37.34 |
| **Small RNA:** | 34462 | 2951726 | 0.16 |
| **Satellites:** | 2953 | 838302 | 0.04 |
| **Simple repeats:** | 580397 | 28765686 | 1.51 |
| **Low complexity:** | 54937 | 4853191 | 0.26 |

**Additional Table 9. Content of repeat elements in the *Anolis carolinensis* genome.**

|  | **Number of elements** | **Length occupied (bp)** | **Percentage of sequence** |
| --- | --- | --- | --- |
| **Retroelements** | 2242332 | 530789914 | 29.5 |
| **SINEs:** | 443355 | 76584647 | 4.26 |
| **Penelope** | 393954 | 57074069 | 3.17 |
| **LINEs:** | 1684725 | 364913695 | 20.28 |
| **CRE/SLACS** | 0 | 0 | 0 |
| **L2/CR1/Rex** | 704649 | 164713219 | 9.16 |
| **R1/LOA/Jockey** | 0 | 0 | 0 |
| **R2/R4/NeSL** | 244595 | 36955059 | 2.05 |
| **RTE/Bov-B** | 223337 | 56970677 | 3.17 |
| **L1/CIN4** | 55123 | 29525681 | 1.64 |
| **LTR elements:** | 114252 | 89291572 | 4.96 |
| **BEL/Pao** | 5173 | 7158627 | 0.4 |
| **Ty1/Copia** | 12320 | 5283274 | 0.29 |
| **Gypsy/DIRS1** | 73032 | 62935021 | 3.5 |
| **Retroviral** | 18936 | 10153865 | 0.56 |
| **DNA transposons** | 861886 | 144912403 | 8.05 |
| **hobo-Activator** | 369164 | 60428226 | 3.36 |
| **Tc1-IS630-Pogo** | 369965 | 68274090 | 3.79 |
| **En-Spm** | 0 | 0 | 0 |
| **MuDR-IS905** | 0 | 0 | 0 |
| **PiggyBac** | 969 | 168940 | 0.01 |
| **Tourist/Harbinger** | 81484 | 9662784 | 0.54 |
| **Other** | 0 | 0 | 0 |
| **Rolling-circles** | 93920 | 24254015 | 1.35 |
| **Unclassified:** | 1506 | 266832 | 0.01 |
| **Total interspersed repeats:** | na | 675969149 | 37.57 |
| **Small RNA:** | 29837 | 2578761 | 0.14 |
| **Satellites:** | 6099 | 1902809 | 0.11 |
| **Simple repeats:** | 492959 | 21208777 | 1.18 |
| **Low complexity:** | 42031 | 2338505 | 0.13 |

**Additional Table 10. Content of repeat elements in *Anolis apletophallus* genome.**

|  | **Number of elements** | **Length occupied (bp)** | **Percentage of sequence** |
| --- | --- | --- | --- |
| **Retroelements** | 1993760 | 395743252 | 18.14 |
| **SINEs:** | 467747 | 70466223 | 3.23 |
| **Penelope** | 182225 | 32773695 | 1.5 |
| **LINEs:** | 1455796 | 300410743 | 13.77 |
| **CRE/SLACS** | 0 | 0 | 0 |
| **L2/CR1/Rex** | 841398 | 162319926 | 7.44 |
| **R1/LOA/Jockey** | 322 | 70651 | 0 |
| **R2/R4/NeSL** | 28895 | 7044990 | 0.32 |
| **RTE/Bov-B** | 261656 | 43587144 | 2 |
| **L1/CIN4** | 92457 | 40869628 | 1.87 |
| **LTR elements:** | 70217 | 24866286 | 1.14 |
| **BEL/Pao** | 3199 | 794646 | 0.04 |
| **Ty1/Copia** | 3479 | 1215599 | 0.06 |
| **Gypsy/DIRS1** | 46237 | 17351451 | 0.8 |
| **Retroviral** | 3198 | 1551805 | 0.07 |
| **DNA transposons** | 1197143 | 144667508 | 6.63 |
| **hobo-Activator** | 674951 | 78820290 | 3.61 |
| **Tc1-IS630-Pogo** | 259669 | 35250873 | 1.62 |
| **En-Spm** | 0 | 0 | 0 |
| **MuDR-IS905** | 0 | 0 | 0 |
| **PiggyBac** | 0 | 0 | 0 |
| **Tourist/Harbinger** | 198545 | 23253126 | 1.07 |
| **Other** | 0 | 0 | 0 |
| **Rolling-circles** | 105448 | 13627326 | 0.62 |
| **Unclassified:** | 1156 | 181522 | 0.01 |
| **Total interspersed repeats:** | na | 540592282 | 24.78 |
| **Small RNA:** | 10052 | 995117 | 0.05 |
| **Satellites:** | 3639 | 1433924 | 0.07 |
| **Simple repeats:** | 616339 | 36363820 | 1.67 |
| **Low complexity:** | 71663 | 7022592 | 0.32 |

**Additional Table 11. Content of repeat elements in *Anolis auratus* genome.**

|  | **Number of elements** | **Length occupied (bp)** | **Percentage of sequence** |
| --- | --- | --- | --- |
| **Retroelements** | 1916123 | 461310730 | 22.86 |
| **SINEs:** | 348524 | 54403272 | 2.7 |
| **Penelope** | 221123 | 39878904 | 1.98 |
| **LINEs:** | 1472571 | 369879139 | 18.33 |
| **CRE/SLACS** | 0 | 0 | 0 |
| **L2/CR1/Rex** | 856048 | 208121218 | 10.32 |
| **R1/LOA/Jockey** | 437 | 107772 | 0.01 |
| **R2/R4/NeSL** | 33915 | 10099122 | 0.5 |
| **RTE/Bov-B** | 215741 | 57679462 | 2.86 |
| **L1/CIN4** | 87788 | 35473264 | 1.76 |
| **LTR elements:** | 95028 | 37028319 | 1.84 |
| **BEL/Pao** | 23666 | 7284004 | 0.36 |
| **Ty1/Copia** | 7757 | 3421414 | 0.17 |
| **Gypsy/DIRS1** | 43706 | 18691097 | 0.93 |
| **Retroviral** | 8676 | 2617411 | 0.13 |
| **DNA transposons** | 970996 | 152701852 | 7.57 |
| **hobo-Activator** | 506939 | 84786861 | 4.2 |
| **Tc1-IS630-Pogo** | 297708 | 46646253 | 2.31 |
| **En-Spm** | 0 | 0 | 0 |
| **MuDR-IS905** | 0 | 0 | 0 |
| **PiggyBac** | 248 | 57277 | 0 |
| **Tourist/Harbinger** | 112533 | 14584867 | 0.72 |
| **Other** | 0 | 0 | 0 |
| **Rolling-circles** | 66825 | 8485753 | 0.42 |
| **Unclassified:** | 904 | 140358 | 0.01 |
| **Total interspersed repeats:** | na | 614152940 | 30.44 |
| **Small RNA:** | 12891 | 1083587 | 0.05 |
| **Satellites:** | 2573 | 189000 | 0.01 |
| **Simple repeats:** | 882277 | 120889949 | 5.99 |
| **Low complexity:** | 70234 | 6324004 | 0.31 |

**Additional Table 12. Run IDs and accession numbers of RNA-seq data used for training of gene prediction on genome assemblies in the Sequence Read Archive of the DNA Data Bank of Japan (DDBJ).**

| **Species** | **Run IDs in the SRA of the DDBJ** | **Accession numbers in the SRA of the DDBJ** |
| --- | --- | --- |
| *Anolis isolepis* | DRR232283 | DRA010304 |
| *Anolis allisoni* | DRR232284 | DRA010304 |
| *Anolis porcatus* | DRR232285 | DRA010304 |
| *Anolis allogus* | DRR055059, DRR055051, DRR055067 | DRA004457 |
| *Anolis homolechis* | DRR055075, DRR055083, DRR055091 | DRA004457 |
| *Anolis sagrei* | DRR055099, DRR055107, DRR055115 | DRA004457 |

**Additional Table 13. Source database of sequence or genome annotation data used in phylogenetic analysis.**

| Species | Genome assembly | The source database of sequence FASTA files and/or GFF file |
| --- | --- | --- |
| *Anas platyrhynchos* | CAU_duck1.0 | Ensemble (release 104) |
| *Anolis apletophallus* | Aapl1.0 | ﻿Harvard Dataverse |
| *Anolis auratus* | Aaur1.0 | ﻿Harvard Dataverse |
| *Anolis carolinensis* | AnoCar2.0 | Ensemble (release 104) |
| *Anolis frenatus* | Afre1.0 | ﻿Harvard Dataverse |
| *Aquila chrysaetos* | bAquChr1.2 | Ensemble (release 104) |
| *Bos taurus* | UOA_Angus_1 | Ensemble (release 104) |
| *Canis lupus* | CanFam3.1 | Ensemble (release 104) |
| *Chelonoidis abingdonii* | ASM359739v1 | Ensemble (release 104) |
| *Chrysemys picta* | Chrysemys_picta_bellii-3.0.3 | Ensemble (release 104) |
| *Crocodylus porosus* | CroPor_comp1 | Ensemble (release 104) |
| *Felis catus* | Felis_catus_9.0 | Ensemble (release 104) |
| *Ficedula albicollis* | FicAlb1.5 | Ensemble (release 104) |
| *Gallus gallus* | GRCg6a | Ensemble (release 104) |
| *Gekko japonicus* | Gekko_japonicus_V1.1 | NCBI |
| *Geospiza fortis* | GeoFor_1.0 | Ensemble (release 104) |
| *Gopherus evgoodei* | rGopEvg1_v1.p | Ensemble (release 104) |
| *Homo sapiens* | GRCh38.p13 | Ensemble (release 104) |
| *Laticauda laticaudata* | latLat_1.0 | Ensemble (release 104) |
| *Latimeria chalumnae* | LatCha1 | Ensemble (release 104) |
| *Meleagris gallopavo* | Turkey_5.1 | Ensemble (release 104) |
| *Monodelphis domestica* | ASM229v1 | Ensemble (release 104) |
| *Mus musculus* | GRCm39 | Ensemble (release 104) |
| *Myotis lucifugus* | Myoluc2.0 | Ensemble (release 104) |
| *Naja naja* | Nana_v5 | Ensemble (release 104) |
| *Notechis scutatus* | TS10Xv2-PRI | Ensemble (release 104) |
| *Ornithorhynchus anatinus* | mOrnAna1.p.v1 | Ensemble (release 104) |
| *Pelodiscus sinensis* | PelSin_1.0 | Ensemble (release 104) |
| *Pogona vitticeps* | pvi1.1 | Ensemble (release 104) |
| *Pseudonaja textilis* | EBS10Xv2-PRI | Ensemble (release 104) |
| *Struthio camelus* | ASM69896v1 | Ensemble (release 104) |
| *Sus scrofa* | Sscrofa11.1 | Ensemble (release 104) |
| *Taeniopygia guttata* | bTaeGut1_v1.p | Ensemble (release 104) |
| *Terrapene carolina* | T_m_triunguis-2.0 | Ensemble (release 104) |
| *Tursiops truncatus* | turTru1 | Ensemble (release 104) |
| *Ursus maritimus* | UrsMar_1.0 | Ensemble (release 104) |

**Additional Fig. 1. The distribution of GC content in 5 kb windows of genome assemblies for *Anolis* lizards after repeat masking.**

**Additional Fig. 2. Gene distribution for each genome assemblies reconstructed in this study.**

**Additional Fig. 3. Repeat landscape for each LINE transposon family of *Anolis* lizards included in this study.**

**Additional Fig. 4. DNA substitution rate (substitutions per billion years) for each branch of phylogenetic tree of sarcopterygian vertebrates.**


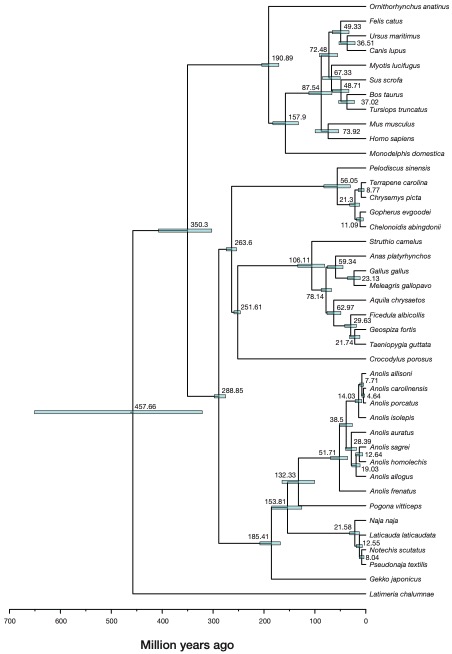


**Additional Fig. 5. Divergence time tree of sarcopterygian vertebrates reconstructed by Bayesian method implemented in MCMCTree.** Node bars indicate 95% highest posterior density.


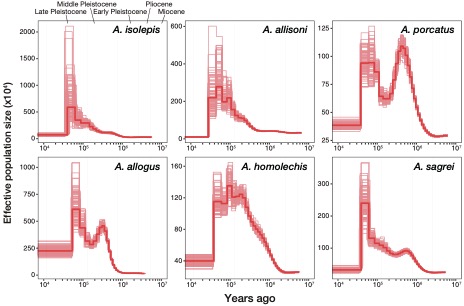


**Additional Fig. 6. Past effective population size of six Cuban *Anolis* lizards inferred using PSMC with bootstrap results.** The thick red lines are the consensus result, and the thin light red lines are each bootstrap result**.**
